# Are HDAC and Glutamine Synthetase Expression Levels Associated with Ga68-DOTATATE PET/CT Data and Prognosis in Gastroenteropancreatic Neuroendocrine Tumours?

**DOI:** 10.3390/medicina61111952

**Published:** 2025-10-30

**Authors:** Ozge Ulas, Ramazan Oguz Yuceer, Zekiye Hasbek, Hatice Ozer, Kerim Seker, Mukaddes Yılmaz, Mahmut Uçar

**Affiliations:** 1 Department of Nuclear Medicine, Faculty of Medicine, Tokat Gaziosmanpasa University, Tokat 60250, Turkey; 2 Department of Pathology, School of Medicine, Sivas Cumhuriyet University, Sivas 58140, Turkey; oguzyuceer@cumhuriyet.edu.tr (R.O.Y.); hozer@cumhuriyet.edu.tr (H.O.); 3 Department of Nuclear Medicine, Faculty of Medicine, Sivas Cumhuriyet University, Sivas 58140, Turkey; zhasbek@cumhuriyet.edu.tr (Z.H.); kerimseker@cumhuriyet.edu.tr (K.S.); 4 Department of Oncology, School of Medicine, Sivas Cumhuriyet University, Sivas 58140, Turkey; ylmzmukaddes@gmail.com (M.Y.); mahmutucar@cumhuriyet.edu.tr (M.U.)

**Keywords:** gastroenteropancreatic neuroendocrine tumours, histone deacetylase enzymes, glutamine synthetase, ^68^Ga-DOTATATE, PET/CT

## Abstract

*Background and Objectives*: Neuroendocrine neoplasms are heterogeneous tumours arising from endocrine gland cells and the neuroendocrine system. Gastroenteropancreatic neuroendocrine tumours (GEP-NETs) constitute two-thirds of this tumour group. This study was aimed at investigating the relationship between histone deacetylase enzymes (HDAC) and glutamine synthetase (GS) positivity, and ^68^Ga-DOTATATE PET/CT data and their effect on prognosis in gastroenteropancreatic neuroendocrine tumours. *Materials and Methods*: Twenty-seven patients with Grade 1 and Grade 2 well-differentiated neuroendocrine tumours, diagnosed by biopsy and admitted to our nuclear medicine clinic for staging were included in the study. *Results*: There was no statistically significant correlation between HDAC and GS positivity in tumours and DOTATATE SUVmax value on PET/CT. There was no significant correlation between HDAC and GS positivity or negativity in the tumour and the presence or absence of metastasis on PET/CT. There was no statistically significant relationship between HDAC and GS positivity and survival. There was a statistically significant correlation between DOTATATE SUVmax value on PET/CT and survival (*p* = 0.039). *Conclusions*: According to the results of the present study, overall survival rates decreased in patients with high ^68^Ga-DOTATATE uptake on PET/CT and therefore, patients with high SUVmax on PET/CT should be followed closely and their prognosis may be poor. In addition, although not statistically significant, the mortality rate is higher in patients with HDAC-positive tumours compared to in patients with HDAC-negative tumours; thus, it should be kept in mind that the prognosis of such patients may also be poor. According to the results of the present study, GS levels were generally negative in NETs. In addition, there was no statistically significant relationship between GS levels and survival.

## 1. Introduction

Neuroendocrine tumours (NETs) are heterogeneous tumours arising from endocrine gland cells and the neuroendocrine system. They constitute approximately 1% of all malignancies. Although they are rare, their prevalence and incidence have continued to increase rapidly in recent years with the impact of diagnostic imaging [1,2]. In recent years, their incidence has increased up to six-fold, reaching a prevalence of 4–5%. NETs are classified according to their origin, invasion, and histologic differentiation [3]. Their grading is based on mitotic count and the Ki-67 index, a nuclear protein marker associated with the rate of cellular proliferation [4]. Tumors with a low or intermediate index are well-differentiated (grade 1 or 2), while grade 3 tumours have a high proliferation index or are poorly differentiated and are classified as neuroendocrine carcinomas [4]. Gastroenteropancreatic neuroendocrine tumours (GEP-NETs) constitute two-thirds of this rare tumour group [5]. Gastroenteropancreatic NETs (GEP-NETs) include tumours arising from the gastrointestinal tract and pancreas. While functional NETs can cause dramatic clinical symptoms due to the overproduction of endogenous hormones or vasoactive substances, non-functional NETs are endocrinologically silent and therefore are usually locally advanced or metastasized at the time of detection. Patients with GEP-NETs usually present with an advanced disease at diagnosis and cannot be treated with surgery alone [6].

The basic mechanism leading to carcinogenesis in NETs is still not fully understood. However, it is known that the mutations found in proto-oncogene and suppressor genes in neuroendocrine tumours are rare in classical tumours, which has led to great interest in epigenetic studies. Epigenetics are reversible changes that occur in the function of genes and cause changes in phenotype without causing changes in DNA sequence. There are many different epigenetic mechanisms in the development of neuroendocrine tumours. Acetylation of histones is one of them. The acetylation status of histones is determined by a reversible balance between histone acetyl transferase (HAT) and histone deacetylase (HDAC) enzymes. In many cancers, this balance between HAT and HDAC activity is disturbed. HDACs exert pro-oncogenic effects by keeping genes that cause differentiation, apoptosis, and cell cycle arrest in a transcriptionally silent state [7]. Since histone deacetylases are abnormally increased in various cancers, they have become the target molecule of cancer research. Histone deacetylase (HDAC) inhibitors facilitate anticancer activity by inhibiting angiogenesis in tumour cells, taking part in the regulation and control of the cell cycle and triggering apoptosis if necessary [8,9]. Therefore, the inhibition of HDACs has been the target of pharmaceutical companies in the development of new anticancer therapy.

Glutamine, which accounts for approximately 20% of the total amino acids in the human body, is the most abundant amino acid in human tissues [10]. It can be supplied either through circulation or synthesized de novo by the enzyme glutamine synthetase (GS), the sole enzyme responsible for endogenous glutamine production [11]. GS catalyzes the conversion of glutamate and ammonia into glutamine in an ATP-dependent manner and is predominantly expressed in the liver, kidneys, skeletal muscles, and brain [12]. In cancer cells, glutamine consumption is markedly increased to sustain proliferation. These cells undergo metabolic reprogramming to support rapid growth and to survive under stressful conditions, such as nutrient deprivation and poor vascularization [13,14]. Because glutamine serves as a critical source of both carbon and nitrogen for cellular biosynthesis, it plays a central role in tumour metabolism. Furthermore, glutaminolysis represents another key metabolic pathway by which cancer cells utilize glutamine to meet their energetic and biosynthetic demands [15,16].

Glutaminolysis is the process of glutamine breakdown catalyzed by glutaminase (GLS) and this process is required to produce energy in various types of cancer. Many cancer cells obtain the glutamine they depend on through glutaminolysis. Therefore, strategies targeting glutaminolysis have been proposed for cancer therapy. However, the metabolic programming differs depending on cancer subtypes. Some cancer types are independent of glutaminolysis for tumour formation and show resistance to glutaminolysis inhibition [17]. In addition, glutaminolysis-resistant cancer subtypes frequently overexpress glutamine synthetase (GS), an enzyme that catalyzes the reverse reaction of glutaminolysis. Cancer cells expressing high GS are self-sufficient for glutamine and can survive in conditions of glutamine deprivation. This study was aimed at investigating the relationship between HDAC and GS positivity, and ^68^Ga-DOTATATE PET/CT data and their effect on prognosis in gastroenteropancreatic neuroendocrine tumours.

## 2. Materials and Methods

The study included twenty-seven patients diagnosed with gastroenteropathic neuroendocrine tumours who were referred to the nuclear medicine department for PET/CT between 2020 and 2024. As the SUVmax value of the primary tumour was to be calculated on PET/CT, the inclusion criteria required histopathological confirmation by biopsy and the absence of prior surgical intervention. In addition, patients who had not received any treatment that could potentially affect the SUVmax value—such as chemotherapy or radiotherapy—were included. Only grade 1–2 well-differentiated tumours with a Ki-67 proliferation index below 20% were eligible for the study. Patients with a second concurrent malignancy were also excluded from the study.

In the Department of Nuclear Medicine, ^68^Ga-DOTATATE PET/CT images were evaluated both visually and quantitatively. The analysis included software-derived standardized uptake value maximum (SUVmax) measurements, the assessment of the presence or absence of metastases, and the documentation of metastatic sites detected on ^68^Ga-DOTATATE PET/CT.

^68^Ga-DOTATATE PET/CT Imaging Protocol: During PET/CT examinations, each patient received an average intravenous dose of 5 mCi (185 MBq) of ^68^Ga-DOTATATE. Following injection, patients rested in a quiet room for 45–60 min to allow for radiotracer uptake. Imaging was performed using a General Electric Discovery PET/CT 600 scanner. The CT component was acquired with a spiral 16-slice scanner operating at 120 kV and 172 mAs to provide attenuation correction and anatomical correlation. Whole-body PET acquisition was performed in three-dimensional mode from the skull base to the proximal thighs, with an acquisition time of approximately 2 min per bed position. Axial, coronal, and sagittal fusion images were reconstructed using an iterative algorithm. SUVmax values were calculated from PET images, applying an adaptive threshold of 42% of the maximum regional activity. The region of interest (ROI) was delineated within the primary tumour.

The following formula was used to calculate the SUVmax:

[Activity in ROI (mCi/mL) × Body Weight (grams)] ÷ Injected Dose (mCi)

### 2.1. Immunohistochemistry

Hematoxylin-and-eosin (H&E)-stained sections, representing the tumours of patients diagnosed with GApnet, were re-examined and paraffin blocks were selected. From these blocks, 4-micron-thick sections were cut and transferred to adhesive-coated slides. Sections were incubated with primary antibodies including HDAC1 (rabbit monoclonal antibody, Clone 10E2, 1:100 dilution, Santa Cruz Biotechnology, Inc., Santa Cruz, CA, USA) and glutamine synthetase (rabbit monoclonal antibody, Clone EPR13022(B), 1:400 dilution, Abcam, Waltham, MA, USA). For confirmation, tissue samples were processed together with positive control tissues; tonsil tissue was used for HDAC1 and liver tissue for glutamine synthetase. All staining procedures were performed using a fully automated immunohistochemistry device (Roche Ventana Benchmark Ultra, Oro Valley, AZ, USA).

### 2.2. Evaluation of Immunohistochemical Staining

Immunohistochemically stained slides were evaluated by two pathologists (R.O.Y., and H:Ö.). Nuclear staining for HDAC1 was considered positive. The intensity of expression was scored as follows: 0, none; 1, weak; 2, moderate; and 3, strong. The extent of staining in the tumour cells was graded as 0 = 0%, 1 = 1–19%, 2 = 20–50%, and 3 = >50%. The H-score was calculated by multiplying the intensity and extent scores. On the basis of the H-score, HDAC1 expression was categorized into the following two groups: negative/low (0–3 points) and high (4–9 points) [18].

For GS, cytoplasmic staining in tumour cells was considered positive. The extent of staining in the tumour cells was graded as 0 = 0%, 1 = 1–19%, 2 = 20–50%, and 3 = >50%. The H-score is calculated by multiplying the intensity and extent scores. On the basis of the H-score, GS expression was categorized into two groups: negative/low (0–3 points), and high (4–6 points) [19].

### 2.3. Statistical Analysis

SPSS version 23 software was used for statistical analysis. The median value was used to express descriptive quantitative data, while percentages were used to express qualitative data. Fisher’s exact test and the chi-square test were used to compare variables. Analytical techniques (Kolmogorov–Smirnov/Shapiro–Wilk tests) and visual methods (histograms and probability graphs) were used to assess whether the variables showed a normal distribution. Descriptive analyses were performed using the median and interquartile range for non-normally distributed variables. When analyzing data that was not normally distributed, the Mann–Whitney U test was employed. The effects of HDAC and GS expression levels and the presence/absence of Ga-68 DOTATATE uptake in the primary tumour on survival in PET/CT were examined using the log rank test. The Kaplan–Meier survival estimates were calculated. Multivariate Cox regression analysis was used to evaluate the independent prognostic factors of GEP-NET. A *p*-value of 0.05 was considered an indication of a statistically significant result.

## 3. Results

The mean age of the participating patients (N = 27) was 57 (26–94) years. Of them, 12 (44.4%) were men, 15 (55.6%) were women, 20 (74.1%) were HDAC-positive and 7 (25.9%) were HDAC negative. In 12 (60%) of the 20 HDAC-positive patients, the tumour showed DOTATATE uptake on PET/CT, whereas no uptake was observed on PET/CT in 8 (40%) patients (Figure S2). DOTATATE uptake on PET/CT was not observed in four (57%) of the seven HDAC-negative patients but was observed in three of them (Figure S3). There was no statistically significant correlation between HDAC positivity in tumours and the DOTATATE SUVmax value in PET/CT (*p* = 0.432). There was no significant correlation between HDAC positivity or negativity in the tumour and the presence or absence of metastasis on PET/CT (*p* = 0.756).

GS was negative in the tumour in 22 of the 27 (81.5%) the patients, and positive in only 5 (18.5%) patients. DOTATATE uptake was seen on PET/CT in three (60%) of the five GS-positive patients, while no uptake was observed on PET/CT in two (40%) patients. DOTATATE uptake on PET/CT was observed in 12 (54.5%) of the 22 GS-negative patients, while no uptake was observed on PET/CT in 10 (45.5%) patients. There was no significant correlation between GS positivity in the tumour and the DOTATATE SUVmax value on PET/CT (*p* = 0.825), and between GS positivity or negativity in the tumour and the presence or absence of metastasis on PET/CT (*p* = 0.726) (Table 1).

**Figure 1:**
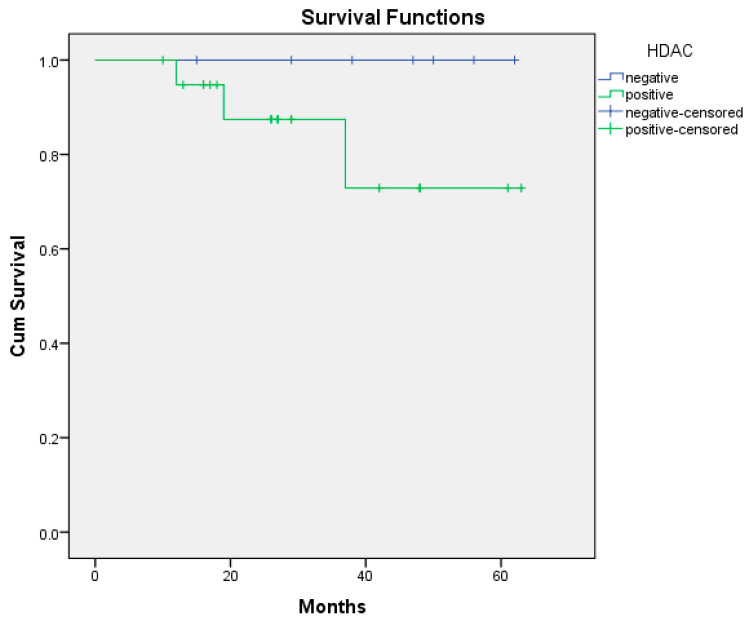
Overall survival curve of HDAC positive/negative (*p* = 0.201). Abbreviations: HDAC: histone deacetylase enzymes.

**Figure 2:**
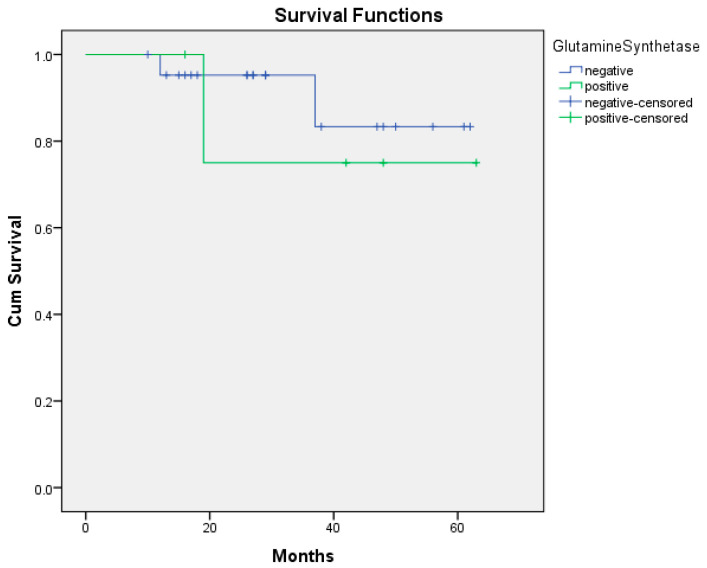
Overall survival curve of GS positive/negative (*p* = 0.653). Abbreviations: GS: glutamine synthetase.

**Figure 3:**
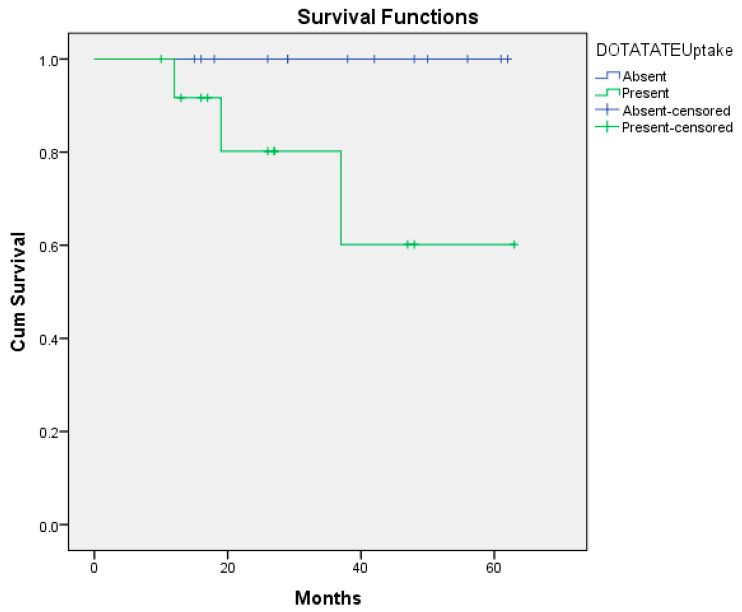
Overall survival curve of DOTATATE uptake positive/negative (*p* = 0.039).

In the 60-month follow-up of the patients, 3 of the 20 HDAC-positive patients died, while none of the 7 HDAC-negative patients died. However, there was no statistically significant relationship between HDAC positivity and survival (*p* = 0.201) (Figure 1). While 1 of the 5 GS-positive patients died, 2 of the 20 GS-negative patients died. There was no statistically significant correlation between GS positivity and survival (*p* = 0.653) (Figure 2). Of the 27 patients, 13 were with DOTATATE uptake on PET/CT (Figure S1). At the 60-month follow-up, of these 13 patients, 3 had died and 10 had survived. None of the 14 patients without DOTATATE uptake on PET/CT died (Figure S3). There was a statistically significant correlation between the presence or absence of DOTATATE uptake on PET/CT and survival (*p* = 0.039) (Figure 3). Based on the 12-month survival analysis, the standard error was 0.051, with a corresponding 95% confidence interval (CI) of 0.792–0.988. For the 19-month survival, the standard error was 0.084, and the 95% CI ranged from 0.672 to 0.959. At 37 months, the standard error increased to 0.151, with a 95% CI of 0.435–0.895. According to the Cox regression analysis, no significant association was observed between HDAC expression, glutamine synthetase positivity, or DOTATATE uptake and prognosis. Specifically, HDAC positivity was not significantly correlated with prognosis in the Cox analysis (*p* = 0.464, HR = 0.024, 95% CI: 0–530.541). According to the Cox regression analysis, no significant association was found between glutamine synthetase positivity and prognosis (*p* = 0.657, HR = 0.579, 95% CI: 0.052–6.44). Similarly, there was no statistically significant correlation between DOTATATE uptake and prognosis (*p* = 0.346, HR = 0.010, 95% CI: 0–142.263).

## 4. Discussion

In the present study, we investigated the expression levels of HDAC and GS enzymes in the cell tissues of GEP-NET patients. According to our results, we found that HDAC enzyme levels were generally positive and GS levels were negative in GEP-NETs. We also compared these enzyme levels with Ga68 DOTATATE PET/CT images and quantitative data. In the literature, we did not find many studies comparable to the present study. However, we found some studies in which HDAC inhibitors were used in NETs and which found that they increased SSTR expression.

To date, the goal of numerous clinical trials has been to improve NET radiation sensitivity through the use of adjunctive therapies. More recently, interest has developed in targeting the epigenetics of NETs to improve radiotherapy sensitivity in these tumours. Recent studies have indicated that the use of HDAC inhibitors increases ^68^Ga-DOTATATE uptake and may improve response to peptide receptor radionuclide therapy (PRRT) treatments. However, in these studies, histone deacetylase inhibitors (HDACi) were administered to all patients regardless of whether the tumours were HDAC-positive or -negative. According to our study, the higher mortality rates, particularly among HDAC-positive patients, led to the hypothesis that HDAC inhibitors might be beneficial if administered to this subgroup. In addition, the results of the present study also revealed that in patients with high ^68^Ga-DOTATATE SUVmax on PET/CT mortality rates statistically significantly increased. However, although the survival analysis yielded a *p*-value of 0.039, the corresponding confidence intervals are not sufficiently robust to confirm the statistical significance of this result. We attribute this limitation primarily to the small sample size, which constitutes the major constraint of our study. Accordingly, we strongly recommend that future studies be conducted with a larger patient cohort to obtain more reliable and generalizable findings.

Although increased SSTR2 expression increases the response to the treatments applied, it seems that the prognosis is poor in these patients.

Recent studies have shown that HDAC inhibitors administered in NETs increase SSTR2 in the tumour [20,21,22,23]. In an in vitro animal experiment study conducted by Guenter et al. using HDAC inhibitors in 38 animals with pancreatic NETs, SSTR2 expression increased in dedifferentiated NETs in ^68^Ga-DOTATATE PET/CT performed before and after the administration of HDAC inhibitors. According to Guenter et al.’s study, the use of ^68^Ga-DOTATATE PET/CT imaging can be increased by using HDACi in patients with pancreatic NET, and the tumour can be targeted for PRRT treatment [24].

In a study in which six HDACi were tested in 3 cell blocks, consisting of pancreatic, mid-bowel, and lung NETs, it was observed that 111In-DOTATATE PET/CT uptake increased significantly after all HDACi treatment in the pancreatic tumour (approximately 8.63-fold after valproic acid), and 111In-DOTATATE uptake increased by an average of 4.45-fold in the pulmonary carcinoid tumour and 2.06-fold in the mid-bowel tumour. According to the results of the same study, HDACi increased SSTR2 expression and 111In-DOTATATE uptake, and it could increase the efficacy of PRRT treatment and could be used together with HDACi [25].

In a study conducted by Pollard et al. on the effect of the use of HDACi with vorinostat on the ^68^Ga-DOTATOC and 123I-MIBG uptake in patients with pancreatic NET that metastasised to the liver, Pollard et al. demonstrated that there was no change in the 123I-MIBG uptake, although a significant increase was observed in the ^68^Ga-DOTATOC uptake [26].

Refardt et al. reported that HDAC given to patients with ^68^Ga-DOTATATE uptake at baseline increased the SSTR2 expression, whereas HDACi did not increase the SSTR2 expression in patients in whom the ^68^Ga-DOTATATE uptake was not observed from baseline onward [27]. Previous studies have administered HDAC inhibitors solely based on SSTR expression, without considering whether the tumours were HDAC-positive or -negative. In our study, although the difference was not statistically significant, SSTR expression levels tended to be higher in HDAC-positive tumours. This finding suggests that the response to PRRT may be more favourable in HDAC-positive tumours due to increased SSTR expression. Further research with larger cohorts is warranted to evaluate the role of HDAC expression in tumour behaviour and therapeutic response.

The Ki-67 proliferation index is one of the most important prognostic indicators in patients with NETs. The Ki-67 proliferation index, one of the most important prognostic indicators in GEP-NETs, is also a key parameter used to determine the tumour grade. In this study, all patients had a Ki-67 proliferation index of less than 20% and were classified as well-differentiated. Consequently, only three patients died during the follow-up period [28]. In the present study, no significant correlation was found between HDAC positivity and prognosis. This may be attributed to the limited sample size, highlighting the need for future studies with larger patient cohorts to further clarify this relationship.

GS has been defined as a basic metabolic enzyme in tumour cells [29,30,31]. GS levels increase especially in patients with hepatocellular carcinoma, skeletal muscle tumours, kidney tumours, papillary thyroid carcinoma, prostate cancer, and breast cancer [32,33,34,35,36]. In addition, in a study of seven patients with GEP-NET tumours, the mechanism of glutaminolysis in NETs was investigated. Glutaminase enzymes were found to be high in GEP-NETs. We believe that metabolic clinical studies with larger patient cohorts are needed to further investigate this issue in NETs.

No significant association was observed between HDAC and glutamine synthetase positivity and prognosis. GEP-NET is a relatively rare condition, and since its incidence is low in our region, the sample size of our study was limited. Consequently, a statistical power analysis could not be performed due to the small number of patients included.

There was also not a significant correlation between GS levels and DOTATATE uptake in tumour cells. It is expected that the present study can contribute to the literature on this subject, because in the literature there is a gap regarding studies conducted that investigate whether GS exists in NETs. In addition, we found only one study in the literature examining the expression of glutaminase in NETs in 7 patients with GEP-NETs [37]. Glutaminase levels were significantly higher in patients with GEP-NETs than in the control group. Perhaps because the glutamine requirement in GEP-NET tumours is met by glutaminolysis rather than GS, the enzyme was found to be low in the present study. We think that new clinical studies with larger patient groups are needed in this regard.

## 5. Conclusions

According to the results of our study, overall survival was reduced in patients with a high ^68^Ga-DOTATATE uptake on PET/CT. Therefore, a poor prognosis should be considered in patients demonstrating high SUVmax values on PET/CT. Only 3 of the 27 patients died within one year. In these patients, the association between high SUVmax levels and the presence of metastatic disease at diagnosis was statistically significant, suggesting a potential link with mortality. Nevertheless, due to the small sample size, this finding should be interpreted with caution and underscores the need for further studies with larger cohorts to validate these results.

In addition, although not statistically significant, the mortality rate is higher in patients with HDAC-positive tumours compared to patients with HDAC-negative tumours; thus, it should be kept in mind that the prognosis of such patients may also be poor. We believe that both clinical and in vitro studies are needed to validate the hypothesis that administering HDAC inhibitors to such patients may enhance treatment response.

Since GS is one of the important metabolic pathways in tumour cells, it has been the target of cancer drug companies. GS levels have been investigated in various cancers, but as far as we know, the relationship between NETs and GS has remained in the background. According to the results of the present study, GS levels were generally negative in NETs. In addition, there was no statistically significant relationship between GS levels and survival.

## Figures and Tables

**Table 1:** Association between HDAC and GS positive/negativity and ^68^Ga DOTATATE uptake.

	HDAC-Positive	HDAC-Negative	GS-Positive	GS-Negative
Positive DOTATATE Uptake	12 (% 80)	3 (% 20)	3 (% 20)	12 (% 80)
Negative DOTATATE Uptake	8 (% 66.7)	4 (% 33.3)	2 (% 16.7)	10 (% 83.3)
Sex				
Female	12 (% 80)	3 (% 20)	3 (% 20)	12 (% 80)
Male	8 (% 66.7)	4 (% 33.3)	2 (% 16.7)	10 (% 83.3)
Age Median (Min–Max)	57.7 (26–94)	52 (45–75)	55 (41–94)	57 (26–76)
Presence of metastasis				
Yes	7 (% 87.5)	1 (% 12.5)	2 (% 25)	6 (% 75)
No	13 (% 68.4)	6 (% 31.6)	3 (% 15.8)	16 (% 84.2)
Which organ				
Duodenum	2 (% 100)	0 (% 0)	0 (% 0)	2 (% 100)
Small intestine	0 (% 0)	1 (% 100)	0 (% 0)	1 (% 100)
Jejenum	1 (% 100)	0 (% 0)	0 (% 0)	1 (% 100)
Colon	1 (% 100)	0 (% 0)	0 (% 0)	1 (% 100)
Gastric	9 (% 75)	3 (% 25)	3 (% 25)	9 (% 75)
Pancreas	4 (% 80)	1 (% 20)	2 (% 40)	3 (% 60)

Abbreviations: HDAC: histone deacetylase enzymes, and GS: glutamine synthetase.

## Data Availability

The original contributions presented in this study are included in the article/Supplementary Materials. Further inquiries can be directed to the corresponding author.

## Supplementary Materials

The following supporting information can be downloaded at: https://www.mdpi.com/article/10.3390/medicina61111952/s1. Figure S1: PET/CT image of a patient diagnosed with liver metastatic pancreatic NET, with ^68^Ga-DOTATATE SUVmax 16 and HDAC positive, GS negative who died within 1 year. Figure S2: (a) Pathology images of the patient in Figure S1 who was GS negative, (b) Pathology images of the HDAC positive patient in Figure S1. Figure S3: PET/CT image of a patient diagnosed with gastric NET who did not show uptake of Ga68 DOTATATE SUVmax and had progression-free survival for 4 years with negative HDAC and GS. Figure S4: (a) Pathology images of the patient in Figure S3 who was GS negative, (b) Pathology images of the HDAC-negative patient in Figure S3.

## Abbreviations

The following abbreviations are used in this manuscript:

GEP-NETs	Gastroenteropancreatic neuroendocrine tumours
HDAC	Histone deacetylase enzymes
GS	Glutamine synthetase
SUVmax	Standard uptake value maksimum
GLS	Glutaminase
PET/CT	Positron emission tomography/Computed tomography
