# Supplementary material for: Are HDAC and Glutamine Synthetase Expression Levels Associated with Ga68-DOTATATE PET/CT Data and Prognosis in Gastroenteropancreatic Neuroendocrine Tumours?
Source: Medicina (Kaunas). 2025 Oct 30;61(11):1952. doi: 10.3390/medicina61111952 (PMC12654112; doi:10.3390/medicina61111952)
Supplement: Supplementary file 1 [file medicina-61-01952-s001.zip › medicina-3895878-supplementary.pdf]

Figure S1. PET/CT image of a patient diagnosed with liver metastatic pancreatic NET, with ^{68}Ga-DOTATATE SUVmax 16 and HDAC positive, GS negative who died within 1 year

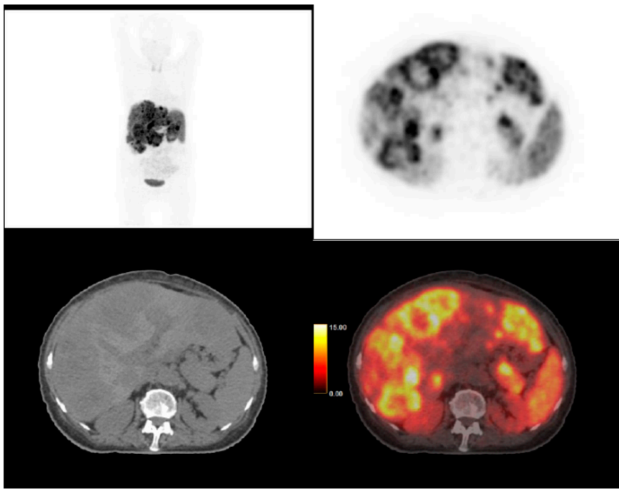

Abbreviations: NET: Neuroendocrine tumor, HDAC: Histone deacetylase enzymes, GS: Glutamine synthetase

Figure S2. **a.** Pathology images of the patient in Figure S1 who was GS negative, **b.** Pathology images of the HDAC positive patient in Figure S1

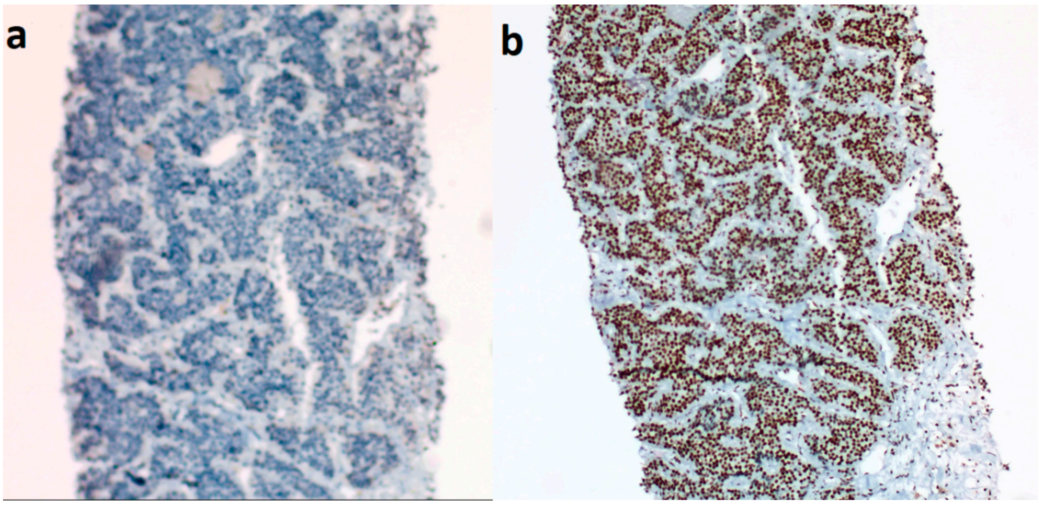

Abbreviations: HDAC: Histone deacetylase enzymes, GS: Glutamine synthetase

Figure S3: PET/CT image of a patient diagnosed with gastric NET who did not show uptake of Ga68 DOTATATE SUVmax and had progression-free survival for 4 years with negative HDAC and GS

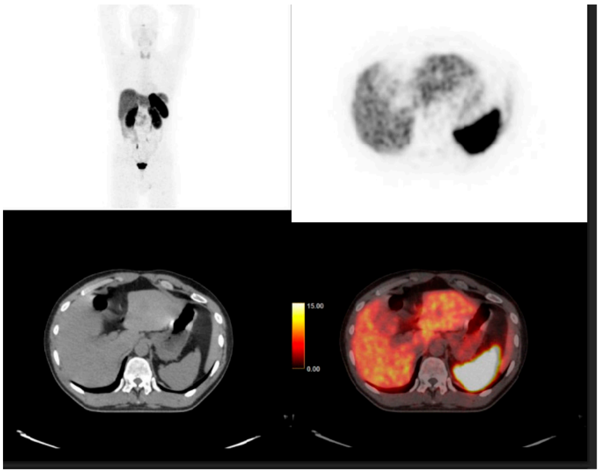

Abbreviations: NET: Neuroendocrine tumor, HDAC: Histone deacetylase enzymes, GS: Glutamine synthetase

Figure S4. a. Pathology images of the patient in Figure S3 who was GS negative, **b.** Pathology images of the HDAC-negative patient in Figure S3

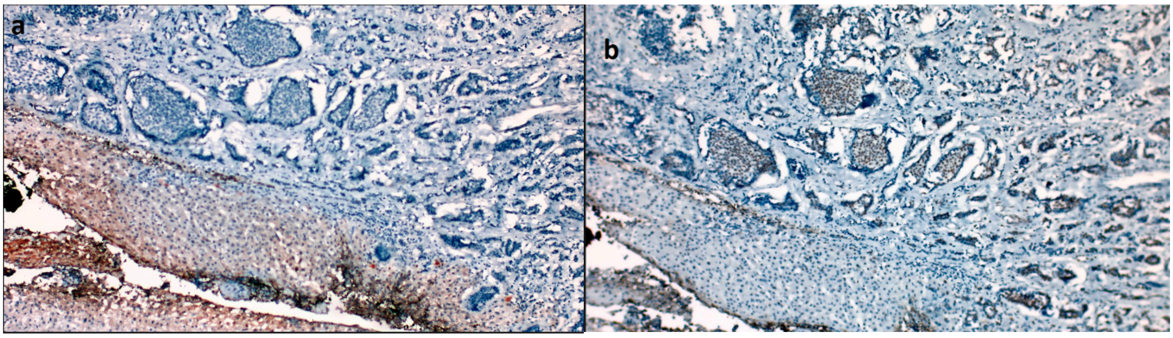

Abbreviations: HDAC: Histone deacetylase enzymes, GS: Glutamine synthetase
